# Barriers and facilitators to institutional delivery in rural areas of Chitwan district, Nepal: a qualitative study

**DOI:** 10.1186/s12978-018-0553-0

**Published:** 2018-06-20

**Authors:** Rajani Shah, Eva A. Rehfuess, Deepak Paudel, Mahesh K. Maskey, Maria Delius

**Affiliations:** 1 Nepal Public Health Foundation, Kathmandu, Nepal; 2 Center for International Health, Ludwig-Maximilians-University, Munich, Germany; 3 Institute for Medical Information Processing, Biometry and Epidemiology, Pettenkofer School of Public Health, Ludwig-Maximilians-University, Munich, Germany; 4 Save the Children, Kathmandu, Nepal; 5 Department of Obstetrics and Gynecology – Campus Grosshadern, Ludwig-Maximilians-University, Munich, Germany

**Keywords:** Qualitative study, Focus group discussion, Qualitative interview, Maternal health, Child birth, Nepal, Access to health services, Quality of health services, Cultural concepts

## Abstract

**Background:**

Giving birth assisted by skilled care in a health facility plays a vital role in preventing maternal deaths. In Nepal, delivery services are free and a cash incentive is provided to women giving birth at a health facility. Nevertheless, about half of women still deliver at home. This study explores socio-cultural and health service-related barriers to and facilitators of institutional delivery.

**Methods:**

Six village development committees in hill and plain areas were selected in Chitwan district. We conducted a total of 10 focus group discussions and 12 in-depth-interviews with relevant stakeholder groups, including mothers, husbands, mothers-in-law, traditional birth attendants, female community health volunteers, health service providers and district health managers. Data were analyzed inductively using thematic analysis.

**Results:**

Three main themes played a role in deciding the place of delivery, i.e. socio-cultural norms and values; access to birthing facilities; and perceptions regarding the quality of health services. Factors encouraging an institutional delivery included complications during labour, supportive husbands and mothers-in-law, the availability of an ambulance, having birthing centres nearby, locally sufficient financial incentives and/or material incentives, the 24-h availability of midwives and friendly health service providers. Socio-cultural barriers to institutional deliveries were deeply held beliefs about childbirth being a normal life event, the wish to be cared for by family members, greater freedom of movement at home, a warm environment, the possibility to obtain appropriate “hot” foods, and shyness of young women and their position in the family hierarchy. Accessibility and quality of health services also presented barriers, including lack of road and transportation, insufficient financial incentives, poor infrastructure and equipment at birthing centres and the young age and perceived incompetence of midwives.

**Conclusion:**

Despite much progress in recent years, this study revealed some important barriers to the utilization of health services. It suggests that a combination of upgrading birthing centres and strengthening the competencies of health personnel while embracing and addressing deeply rooted family values and traditions can improve existing programmes and further increase institutional delivery rates.

## Plain English summary

Giving birth at a health facility plays an important role in preventing maternal deaths. Although child birth services are free and women receive a cash incentive for giving birth at a health facility in Nepal, about half of women give birth at home. This study was conducted to identify the socio-cultural and health service factors influencing birth at health facility. Altogether, 10 group discussions and 12 interviews were conducted in plain and hill areas of Chitwan district of Nepal to collect data from mothers, their husbands and mothers-in-law, community members, service providers and health managers.

The factors that encouraged birth at a health facility were danger signs in giving birth, supportive husbands and mothers-in-law, availability of transport, a birthing facility located close to home, a sufficient cash incentive, as well as the availability and friendliness of health workers. The socio-cultural factors hindering birth at a health facility were the fact that child birth is perceived as a normal event, care available from family members, freedom of movement and a warm environment and appropriate foods being available at home, pregnant women’s shyness and position in the family, the lack of roads and appropriate transportation, an insufficient cash incentive, the poor condition of birthing centres, including lack of equipment, and the young age and perceived lack of competence of health service provider and viewing service provider not competent.

Making improvements to birthing centres, enhancing the skills of service providers, and addressing family values and traditions are necessary to increase births at health facilities in Chitwan district.

## Background

The maternal mortality ratio (MMR) in Nepal has decreased by 71% between the years 1990 and 2015 compared with an average reduction of 67% in Southern Asian countries during the same period. Nevertheless, the MMR (258 deaths per 100,000 live births) in Nepal remains higher than in all other Southern Asian countries except for Afghanistan [1].

Giving birth assisted by a skilled birth attendant in a health facility plays a vital role in preventing maternal deaths [2, 3]. Fifteen percent of pregnancies are associated with critical complications and unpredictable consequences and thus require access to emergency obstetric care [4]. Even an uncomplicated childbirth requires skilled care and the continuous presence of a health professional [5].

To encourage institutional delivery in Nepal, delivery services have been provided free of charge at government health facilities as well as selected private facilities [6] since 2009. To help overcome the country-specific transportation challenges and associated transportation costs – Nepal is characterized by three distinct geographical terrains, i.e. plain areas, hill areas and mountain areas –a cash incentive for institutional deliveries was introduced by the national maternal healthcare programme called Aama in 2005 [7]. This amounts to Nepali Rupees (NRs) 500 ($5), 1000 ($10) and 1500 ($15) in plain, hill and mountain districts respectively. Since 2012, an additional NRs 400 ($4) has been provided for completing four antenatal care (ANC) visits according to schedule to pregnant mothers across all of the country [8].

Nevertheless, only 57% of births take place in a health facility [9]. Several studies have quantitatively examined the factors contributing to the low institutional delivery rate in Nepal and have provided useful insight [10–17]. A few qualitative studies have also been published in the Nepalese context [18, 19]. However, a more comprehensive understanding of the problem, which takes into account the perspectives of all those directly involved with the decision regarding the place of delivery and associated care, is much needed. By employing a qualitative research design, using both focus group discussions (FGDs) and in-depth interviews (IDIs), the present study attempts to achieve deeper insights into the problem and to identify useful pointers to solutions. It aims to obtain in-depth information on socio-cultural and health service-related barriers to and facilitators of institutional delivery from the perspectives of family and community members as well as health service providers at community level, and district health managers.

## Methods

### Study setting

Nepal is characterized by plain, hill and mountain ecological regions. As per the new constitution of Nepal issued in 2015, Nepal has been administratively restructured into 7 provinces and 77 districts; the latter are further divided into urban and rural municipalities [20]. Before restructuring, each of the 75 districts comprised rural village development committees (VDCs) and urban municipalities. Each VDC and municipality was further divided into smaller administrative units called wards [21]. The rural municipalities have been established through merging the previously existent VDCs.

Chitwan district is classified as a plain district but consists of both hill and plain areas. At the time of data collection, Chitwan had a population of approximately 580,000 [22]. It is among the six districts with the highest (> 0.550) human development index [23] One third of males in the district do not live with their families as they work elsewhere. Around 73% of females are married by 19 years of age and then live with their husbands’ family [24].

In Chitwan, there are three major referral hospitals in the district headquarter, Bharatpur, among several other private hospitals with birthing facilities. Health services in rural areas are provided through one hospital, three primary health care centers, 38 health posts and two community health units (District Public Health Office: Birthing centres in Chitwan district 2017, unpublished data). At the time of data collection, a total of 14 birthing centres operated 24 h 365 days a year in 14 VDCs. Birthing centres are established at health facilities by assigning a midwife – i.e. an auxiliary nurse midwife (ANM) with 18-months of midwifery training – that can assist uncomplicated births [25]. In addition, each ward has at least one female community health volunteer (FCHV) to promote the utilization of maternal, child and reproductive health services [8]. While official reports show that 83% of deliveries in Chitwan district take place in health facilities, this masks a difference between rural and urban areas, with several rural areas, in both plain and hill regions showing much lower institutional delivery rates [26]. The geographically relatively advantaged situation of Chitwan, where most inhabitants live within reach of a health institution suggests that access to health services is not the predominant reason for choosing to deliver at home. Therefore, the district is particularly well-suited to explore the range of factors influencing the choice of place of delivery because women, in principle, have the opportunity to give birth at a health facility.

For this study, we purposively selected VDCs where the percentage of institutional deliveries was below the average for the district, including three VDCs in hill areas, i.e. Chandivanjyang (15%), Kabilas (41%) and Kaule (7%), and three VDCs in plain areas, i.e. Padampur (78%), Piple (67%) and Ayodhyapuri (68%) (District Public Health Office: Data on maternal health in Chitwan 2010/2011, unpublished data); four of these VDCs had a birthing centre (Table 1). The population in these VDCs comprises 67% Janajatis, 25% Brahmans/Chhetris, 8% Dalits and less than 1% Muslims [27].

**Table 1:** Data collection through focus group discussions and in-depth interviews

	Plain areas			Hill areas			Total
	Ayodhyapuri*	Piple*	Padampur	Chandi Vanjyang*	Kaule*	Kabilas	
Focus group discussions							
Mothers with home delivery		1	1	1		1	4
Mothers with institutional delivery		1	1		1	1	4
Mothers-in-law	1				1		2
In-depth interviews							
Husbands		1			1		2
TBAs	1				1		2
FCHVs	1			1			2
Service providers							
- In-charge of health post		1			1		2
- ANM	1			1			2
District health managers							
- DPHO	1						
- Safe motherhood focal person	1						

*with birthing centre

*TBA* Traditional birth attendant; *FCHV* Female community health volunteer; *ANM* Auxiliary nurse midwife; *DPHO* District public health officer

### Study design and study participants

Mothers who had given birth between May 2011 and April 2012 were the focus of this study. We conducted 10 focus group discussions (FGDs) and 12 in-depth-interviews (IDIs) [28, 29] with relevant stakeholder groups (Table 1). For the FGDs we purposively selected – with the help of FCHVs – mothers who delivered at home, mothers who delivered at a health institution and their mothers-in-law. Participants for FGDs were identified according to the following criteria: mothers who had given birth in the year preceding the survey, residency in the study area during the time of delivery and willingness to take part in the study. In addition, we conducted IDIs with husbands, traditional birth attendants (TBAs), FCHVs and health workers (service providers), i.e. the in-charge and the ANM of the local health post. Moreover, we interviewed two district health managers: the district public health officer (DPHO) and the focal person for safe motherhood. Data triangulation by comparing and contrasting the perspectives of different groups of study participants as well as involving different approaches to data collection – i.e. FGDs as well as in-depth interviews helped to consolidate the findings on enabling factors for and barriers to institutional delivery [30].

### Data collection

Data collection was carried out between May and August 2012. The FGDs were conducted in a classroom of the local school during holidays or in a room inside a community building; the IDIs variably took place in a closed room inside a health facility, district health office, community building or the home of participants. The duration of FGDs ranged from 65 to 90 min, while the IDIs ranged from 45 to 75 min.

FGD and IDI guides, available upon request, were prepared on the basis of available literature [31–36]; their structure is depicted in Table 2. Probing questions were also formulated. After pre-testing the guides [37] with three FGDs in Piple VDC in areas that were not part of this study, minor modifications were made, such as changing the order of questions and rewording selected questions.

**Table 2:** Overall structure of the FGD and IDI guides

• Status of maternal health • Trends in maternal care seeking behaviours • Common practices before delivery and underlying reasons • Common practices during delivery and underlying reasons • Enabling factors contributing to institutional delivery • Barriers to institutional delivery • Roles of family members • Roles of community members, i.e. TBAs, FCHVs, neighbours • Opinions about birthing centres available in the community • Suggestions to increase institutional delivery	

Data collection was carried out by RS in the Nepali language. All FGDs and IDIs were audio taped. Two Bachelor of Public Health students were trained and then, one at a time, helped with taking notes. All the data were transcribed from the audio recordings and notes. The transcripts were subsequently translated by RS into English.

### Data analysis

Data were analyzed manually using thematic analysis [38]. Initially, the transcripts were read multiple times for familiarization with the data. Coding was done by RS and Jukki Chaudhary - a public health lecturer. Both coded the same two FGDs and three IDIs independently. The codes identified from the texts were then discussed and final codes decided, with input from MD. Using the final codes, RS then coded all the remaining transcripts [39], starting off with the FGDs with the mothers, followed by analysis of the FGDs with mothers-in-law and IDIs with husbands, community informants and service providers; the IDIs with the district level managers were analysed at the end. Following line-by-line coding, codes with similar meanings were grouped together to form a common theme. The themes were then reviewed by both researchers and some themes combined into a broader single theme with relevant sub-themes. Similarities or contrasting views of study participants were compared. Finally, the connections or relations between different themes as well as sub-themes were explained to identify the factors influencing institutional delivery in positive or negative ways. This last stage of the process was completed in an iterative manner through regular discussions between all members of the research team.

### Ethical consideration

Ethical approval for the study was obtained from the Nepal Health Research Council (registration number 21/2012). Permission to conduct the study was obtained from the district public health office, Chitwan. Informed consent was obtained from the participants themselves or their parents, guardians or next of kin for participating mothers below the legal age; as many participants were illiterate, in particular mothers, informed consent was often obtained verbally rather than in writing. Voluntary participation was ensured throughout the study. All information collected was kept confidential. Personal identifiers were removed from the records after data analysis, and the analysis did not contain any identifying information.

## Results

Socio-demographic characteristics differed between women who had given birth at home and women who had delivered in a health institution. Women who had delivered at home were older and of higher parity; all of them were from disadvantaged castes and the majority had only primary level education or no schooling at all (Table 3). Almost all mothers-in-law were illiterate; the majority belonged to a disadvantaged caste. The participating husbands were also from disadvantaged caste and were involved in both agriculture and labour work. The TBAs and FCHVs belonged to both disadvantaged and advantaged castes. The remaining six IDIs were conducted with ANMs and in-charge of the local health facility and district level managers.

**Table 3:** Socio-demographic characteristics of mothers participating in FGDs

	Plain VDCs		Hill VDCs		Total
Place of delivery	Institutional delivery (*n* = 12)	Home Delivery (*n* = 12)	Institutional delivery (*n* = 12)	Home Delivery (*n* = 14)	
Age					
Up to 19 years	3	2	3	3	11
20–29 years	9	9	8	9	35
30 and above	0	1	1	2	4
Parity					
1st	7	5	7	5	24
2nd or more	5	7	5	9	26
Caste / ethnicity					
Disadvantaged*	11	12	8	13	44
Advantaged**	1	0	4	1	6
Educational status					
None or primary	5	6	4	12	27
Secondary or higher	7	6	8	2	23

*Disadvantaged: Dalits and disadvantaged Janjati (Chepang, Rai, Magar, Tamang, Darai, Chaudhary)

**Advantaged: Gurung, Newar, Brahman, Chhetri

During analysis, three major themes influencing the place of delivery emerged: socio-cultural norms and values, comprising traditional care during birth and the post-partum period and family hierarchy and social norms as important sub-themes; access to birthing facilities; and perceptions regarding the quality of health services. For each of these major themes, the arguments in favour or against institutional delivery varied according to people’s background and where they live. Table 4 provides an overview of the insights gained regarding facilitators and barriers to institutional delivery. More detailed insights are provided in the following sections.

**Table 4:** Facilitators of and barriers to institutional delivery

Themes	Sub-themes	Facilitators	Barriers
Socio-cultural norms and values	Traditional care during birth and the post-partum period	• Institutional delivery in case of complications	• Childbirth as a normal life event • Care by family members, neighbours and TBAs • Freedom of movement during birth • Warm environment after birth • Food choices and practices
	Family hierarchy and social norms	• Husbands and parents-in-law supportive of institutional delivery	• Shyness • Low caste, poor education, early marriage • Husbands and parents-in-law not supportive of institutional delivery
Access to birthing facilities		• Ambulance available • Birthing centre nearby • Sufficient financial incentives • Material incentives: clothes for mother and baby	• Lack of roads (hill areas) or good roads • Distance from health institution (especially in hill areas) • Ambulance not always available • Insufficient incentives
Perceptions regarding the quality of health services		• 24-h availability of midwives • Friendliness of health workers	• Perceived incompetence of midwives • Young age of midwives • Poor infrastructure and lack of equipment at birthing centres • Low budget allocated to birthing centres

*TBA* Traditional birth attendant

### Socio-cultural norms and values

#### Traditional care during birth and the post-partum period

Many women considered giving birth to be a normal life event and believed that in the absence of complications there is no need for an institutional delivery. Even those living very close to a health post did not consider institutional delivery necessary, as they felt that they could easily obtain help at home in case of complications. In some plain areas, where TBAs were still active, some mothers and family members indicated that they preferred to be assisted by a TBA. Consulting a traditional healer for delivery was, however, not usually practiced. Very few trusted the traditional healers’ estimates of birthing time and sought their help for identifying whether the pain was due to supernatural evil forces or not. Generally, in hill and plain areas, most of the mothers, family members and TBAs believed that women should be taken to a birthing facility in case of complications, such as heavy bleeding, or after prolonged labour, which was described as continuous labour lasting for 24 h in plain areas and lasting for 72 h in hill areas.

*“I think the main reason for not going to a health facility is that women think delivery is a normal event. Women hear mothers-in-law and mothers saying – ‘we delivered more than 12 babies while cutting grass, cut the cord with sickle and put baby on lap’.”* (FCHV, hill VDC).

*“My wife had no complication to go to the health post. Even in case of complications, we have health workers of the health post always available to assist in home deliveries.”* (Husband, home delivery, hill VDC).

*“If TBA can assist the birth, she says it will happen after some time. Then we don’t need to take her (the mother) to the health institution. If she cannot conduct delivery, she would ask to take her to the health institution as soon as possible.”* (Mother-in-law, home delivery, plain VDC).

*“If a woman cannot give birth even after two to three days, then we must take her to a health institution. Yesterday the woman who went to the health post for delivery was also suffering from labour for three days.”* (Mother, home delivery, hill VDC).

Mothers as well as their husbands reported that the care received from family members and neighbours during child birth was an important reason for their preference to deliver at home. The mothers – and, after birth, the children, too – were massaged with mustard oil. The possibility of free movement during birth was also seen as a clear advantage.

“*At home, we have people holding our hands, legs and body, doing massage on them, and encouraging and giving comfort during labour and childbirth. In the hospital, no-one cares at all. Doctors come only at the time of delivery, they don’t come earlier to listen to our problems.*” (Mother, home delivery, plain VDC).

*“Women’s movement and position during labour is restricted at health institutions. Women must keep sitting or lying in bed at the health facility. There are more difficulties at a health institution than at home.”* (Husband, home delivery, hill VDC).

After birth, the mother and her baby traditionally rest in a corner of the house. Since post-partum women are considered to be in a “cold” state, “hot” foods (where hot does not necessarily refer to temperature or spices) and a warm environment (usually a fire) are believed to be necessary to maintain health and regain strength. Consequently, food practices dictate that the mother is given special nutritious food that is categorized as hot and soft. Many mothers who gave birth at home mentioned that the opportunities to have hot foods immediately after birth and to keep the mother and newborn in a warm environment motivated them to give birth at home.

*“After delivery women should stay in the health post until the bleeding stops. It is cold in the health post and the baby can die due to hypothermia. They have the culture of staying in a corner of their house for many days after childbirth.”* (FCHV, hill VDC).

*“There are no warm beds/clothes for mothers and no room with warm environment such as a heating facility to keep the baby warm after birth.”* (Husband, home delivery, hill VDC).

*“Hot foods are available at home immediately after birth, at the hospital who would give us? People do not have money to buy food at a hotel.”* (Mother, home delivery, plain VDC).

*“The expenses to take a woman to hospital could be used for her foods for postpartum period.”* (Mother, home delivery, plain VDC).

#### Family hierarchy and social norms

Shyness emerged as an important theme. Many mothers stated clearly that an important reason for not wanting to deliver at a health institution was their being shy to show their genitals to others, in particular to male doctors. Chepang mothers- a disad

taged Janajati caste- were reported to be even more timid than women belonging to other castes.

*“I feel shy to show my genitals to others, and even to a female nurse.”* (Mother, home delivery, plain VDC).

*“Chepang women are very shy, even some mothers-in-law are shy. They say that they will not show their genitals to anyone. … And women would prefer to deliver in the corner of their house.”* (Mother-in-law, institutional delivery, hill VDC).

Some mothers and FCHVs indicated that the mothers did not dare to talk with their family members about the option of institutional delivery. This perspective was shared by mothers-in-law, some of whom stated that there was a lack of communication between them and their daughters-in-law. A midwife attributed women’s hesitation to speak openly to early marriage and low education.

*“Pregnant women want to come to the health post, but they cannot say this to their family members. They feel shy and awkward to talk to family members about the place of delivery. They say it at the last stage, if they are unable to deliver the baby.”* (Mother, home delivery, hill VDC).

*“My daughters-in-law didn’t give any information about their labour. How would I know their condition unless they tell me*?” (Mother-in-law, home delivery, hill VDC).

Within the family hierarchy, decisions are made by or in accordance with the husband and the parents-in-law. Some women reported that mothers-in-law were the decision makers in the family and that fathers-in-law generally supported their views. Often this led to an institutional delivery, although at times it could be the opposite, depending on the experiences and beliefs of different family members. Nevertheless, most of the women who delivered at home indicated that they had chosen to do so themselves rather than being persuaded by family members. Most women who had given birth at a health institution stated that their husbands had encouraged and supported their choice.

*“We didn’t go because of our confidence in being able to give birth at home, not due to pressure of our family members”* (Mother, home delivery, plain VDC).

*“My husband was willing to take me to the hospital, but my mother-in-law said it can be done at home. My husband was very afraid at first.”* (Mother, home delivery, plain VDC).

### Access to birthing facilities

Despite the relatively large number of birthing centres in Chitwan district, physical access is still a challenge in some areas, especially where birthing centres are located far from a pregnant woman’s home. Mothers living in hill areas faced greater difficulties in reaching a health facility than women living in the plain due to lack of roads and transportation. In fact, in a few places, women had to be carried in a bamboo basket, hammock or stretcher; when husbands were not at home and other men not available this constituted an important barrier. Some respondents were afraid of having an accident on the way to the health facility.

*“We have taken a bamboo basket from the health post. It’s difficult to carry a woman alone in a bamboo basket. We had kept a stretcher too, but a pregnant woman nearly died falling out of a stretcher. Roads are narrow, sloppy and steep.”* (FCHV, hill VDC).

*“Men cannot be found in the village to carry a pregnant woman to the health facility. They are in other districts for work”* (Mother, home delivery, hill VDC).

In contrast, in the plain, the ambulance was often readily available; however, some mothers reported that it can take a long time for the ambulance to arrive. Difficulty in travelling due to poor road condition was also reported by some of the mothers living in plain areas.

*“Ambulance comes immediately after we call. Other public vehicles are available in five minutes.”* (Mother, institutional delivery, plain VDC).

*“When we called the ambulance, it had gone to drop another patient, so it was late. Labour pain occurred only for three hours. Delivery happened before the arrival of the ambulance.”* (Mother, home delivery, plain VDC).

*“If we go to hospital, there would be jerking in the bus because of poor roads, our body shakes and blood moves. It’s convenient to give birth at home.”* (Mother, home delivery, plain VDC).

Almost all participants were aware of the cash incentives for institutional delivery provided by the government. Some mothers and their family members reported that relatively little money was required for delivery at the nearest birthing centre. Many others, however, indicated that the incentive provided was not sufficient to meet all of the expenses related to an institutional delivery, such as transportation fare, cost of food during the stay at the health facility and additional food costs for those carrying the woman to the health facility in hill areas.

*“As foods can also be taken from home, a lot of money is saved if we go to the nearest birthing centre. NRs. 100 is enough for delivery here. People with low economic status are going to the local birthing centre.”* (Mother, institutional delivery, plain VDC).

*“Community people ask to go to health facility but a lot of money is necessary for hiring the ambulance, food in hospital, providing food to the people who carry the mothers, etc.; a small sum of incentive provided is like a drop in the ocean.”*(Mother, institutional delivery, hill VDC).

*“Many people are required to carry a woman to a health facility. Those who carry need to be provided with alcohol and other snacks in the hotel. Postpartum mothers need ‘Jaulo’- a soft food made from rice and pulse, soon after birth. About NRs. 2000 is needed. So, they don’t care about NRs. 900 provided by the health facility.”* (FCHV, hill VDC).

*“We would expect hot soup and nutritious food provided to the mother after birth, in addition to the cash being provided*.” (Mother, institutional delivery, hill VDC).

However, FCHVs of both hill and plain areas believed that the lack of money was not the main reason for delivering at home. In fact, some respondents suggested that a material incentive was valued more than a monetary incentive because the gifts could be used by the mother and the newborn themselves.

*“Money is not the reason for not going to health institution. When a traditional healer comes they immediately sacrifice rooster to serve him and give him at least NRs. 500 for helping them.”* (FCHV, hill VDC).

*“The money we give may not reach the hand of the mother, the husband may spend it on drinking or the mother-in-law may use it for smoking. A blanket for the mother and clothes for the newborn provided by a non-governmental organization have impressed the mothers more. The number of health facility deliveries has increased significantly in the last two months since provision with the clothes started”* (Service provider at a health post, hill VDC).

### Perceptions regarding the quality of health services

Supported by recent government programmes many new birthing centres have been set up over a relatively short period of time. The quality of the infrastructure, including the lack of a warm environment, and the availability of the necessary equipment, especially in relation to complicated deliveries, as well as the competence of health personnel were put into question by many local people. Family members, FCHVs and health workers also reported that there was a problem in accommodating mothers and their accompanying family members. Consequently, women able to access health facilities, tended to prefer hospitals in the district headquarter Bharatpur to local birthing centres. One of the district health managers attributed these infrastructure problems to the low budget allocated by the government.

*“There is no waiting room, there is a problem in maintaining privacy due to lack of partition of the room, the delivery bed is inappropriate, and the available one is not in good condition*.” (Service provider at a health post, hill VDC).

*“If women need to do surgery, they don’t have the required equipment and instruments at the health post. So, people are not assured of the service provided from the health post.”* (Mother, home delivery, hill VDC).

*“Those of higher economic status go to a hospital. Some of them don’t have trust in the services of the health post. Middle and lower class women visit the local health facility for delivery*.” (Service provider at a health post, plain VDC).

*“NRs. 100000 ($1000) is provided for the opening of two birthing centres per year. Managing the infrastructure, beds, and other materials with fifty thousand is very difficult. Only a delivery bed costs thirty to forty thousand rupees.”* (District health manager).

Concern was also expressed about the competence of the midwives at the new birthing centres. Most of the mothers and family members who had not visited the nearest birthing facility justified this by considering the midwives at the facility as not sufficiently competent to deliver care or manage referrals appropriately. Their opinion was shared by FCHVs, who added that these concerns were largely attributable to the midwives being very young. In fact, many of the midwives themselves felt uncertain about their competence due to their lack of skilled birth attendant training and unavailability of materials.

*“Some people say that ANMs are young and are here to learn. Small girls cannot perform well.”* (FCHV, plain VDC).

*“We have not received SBA training, so, we lack skills to manage complicated cases. We lack materials even like epi set, placenta bowel, hand washing pot, etc.”* (Service provider at a health post, plain VDC).

On the other hand, some mothers and FCHVs praised the fact that midwives were available day and night, and regarded midwives to be both friendly and caring. This positive behaviour by health personnel towards patients and visitors was reported to encourage use of health facilities.

*“Midwives at nearby birthing centre do not get angry and teach good things*.” (Mother, institutional delivery, plain VDC).

*“People might have heard about better services being provided by the health facilities than before, and the people might have gone to the facilities due to the love/affection, proper care and good behavior of the health workers towards the people*.” (FCHV, plain VDC).

## Discussion

This qualitative study identified a broad range of issues that influence place of delivery. Three major themes – socio-cultural norms and values (with the sub-themes traditional care during birth and the post-partum period and family hierarchy and social norms), access to birthing facilities, and perceptions regarding the quality of health services – emerged. Importantly, the analysis combined insights obtained through FGDs and IDIs conducted with mothers who had recently given birth, different family members and distinct health personnel involved with care during pregnancy, childbirth and the post-partum period offering overlapping and at times contrasting perspectives. In the following sections, findings and perspectives identified through the present study are compared with those already available in the literature for Nepal and other low- and middle-income countries.

In contrast to several previously undertaken quantitative studies in Nepal, we believe that this qualitative study adds value by providing in-depth insights into issues that local people care about deeply. Such issues, in particular socio-cultural aspects that vary much across cultures, cannot be easily assessed through a quantitative survey. In addition, such issues can easily be dismissed as they are perceived to be “normal” within a society and thus may not be articulated. Since this study was carried out in an area with relatively good access to health facilities and a relatively high institutional delivery rate it is especially suited to explore underlying reasons other than infrastructural problems, which still constitute a main reason for not using health facilities in many other parts of Nepal.

### Socio-cultural norms and values

#### Traditional care during birth and the post-partum period

In this study, most of the mothers and their family members believed that an institutional delivery was necessary if complications occurred during childbirth. In contrast, with uncomplicated cases, some mothers considered it to be unnecessary, having seen their own mothers and mothers-in-law give birth at home. This is consistent with the findings of a community-based study in Nepal where about one third of mothers did object to the necessity of an institutional delivery [11]. In another study in Nepal, having experienced or witnessed an uncomplicated home delivery was a major reason for rejecting institutional delivery [40]. Similarly, in two studies from Indonesia and Ethiopia childbirth was perceived as a natural phenomenon. Seeking help from a health service provider rather than a TBA was only considered necessary in complicated cases [35, 41].

In addition, this study found that Nepali cultural practices during and after childbirth, such as being able to move freely during labour, staying in a warm environment, having special foods and enjoying traditional massages, were important reasons for giving birth at home. Freedom of movement and care by family members during delivery were previously articulated as reasons for home delivery in Nepal [18]. A more in-depth study of cultural practices and beliefs about childbirth and the post-partum period in Nepal identified rituals regarding the delivery of the placenta and cord cutting, seclusion of and care for women after birth, purification and naming ceremonies and food practices, and stressed that these rituals influence the use of health services [42]. In rural Laos home deliveries were preferred for the emotional and physical care provided by husbands and other family members during labour, including back massages and gentle touching of the belly [43]. Similarly, providing warmth to mothers by locating them close to a coal fire was cited as one of the reasons for choosing home deliveries in Ethiopia [44]. Many different rituals in the post-partum period are found around the globe. The mother is often confined to a specific place, sometimes because she is considered to be impure. Food rules and taboos, while varying greatly between regions, appear to be universally important [45].

#### Family hierarchy and social norms

In the patrilocal system of Nepal, after marriage women move to the home of their husbands’ family; many of them are very young when giving birth to their first child. Within the traditional family hierarchy young mothers have a subordinate position, they tend to be very shy and usually follow the decisions made by other family members [18, 33, 46].

In our study, most mothers reported that their husbands generally supported an institutional delivery but many women were only taken to a birthing facility when complications occurred. Similar findings were obtained in a quantitative study undertaken in the same area [13]. A previous study in Nepal reported that husbands played a cardinal role in the decision to give birth at a health facility [47]. Similarly, in rural Bangladesh, those women delivering at a health institution had husbands who had set aside money and arranged for transportation [48]. Several factors prevented husbands in Nepal from supporting their wives during pregnancy, birth and the post-partum period: lack of knowledge about their role in childbirth, social stigma, embarrassment and job responsibilities [49]. In Uganda, husbands accompanying their wives in cases of severe obstetric complications reported that they lacked information about their role during childbirth; unfriendly health personnel further confused them [50].

In the current study, most mothers-in-law supported an institutional delivery, while some were opposed to it, mainly because they did not consider it essential; shyness in communication between pregnant women and their mothers-in-law may have contributed to this. Another study conducted in Nepal estimated that in 13% of pregnancies the mother-in-law forbade an institutional delivery, because she considered it to be unnecessary and too expensive [11]. Similar reasons were described when ANC use was not supported by mothers-in-law [51]. In addition to feeling shy in communicating with other family members, pregnant women consulted in our study hesitated to go to a health facility because they feared lack of privacy, coupled with a feeling of timidity. Shyness and fear of institutional delivery were also cited in another study in Nepal [11] as well as in a study in rural Laos [43].

### Access to birthing facilities

Physical access to a birthing facility is determined by a combination of distance from the health facility and availability of transport. In the current study, many participants indicated that due to the establishment of birthing centres at health posts in remote areas and the reliable availability of an ambulance, access had improved, even for poor and disadvantaged women. In another study in the hill areas of Nepal the lack of roads was shown to be an important barrier to reaching the birthing facility [18]. Other studies in Nepal indicated that women who could reach a birthing facility within one hour were more likely to have an institutional delivery than those who took longer to get there [10–13, 15]. Consistent results were observed in rural Zambia [52], Indonesia [35] and rural Laos [43].

Although the significance assigned to an institutional delivery (or lack thereof) played a greater role than financial means, money was nevertheless of concern for many people, especially in hill areas. The government incentive was reported to be inadequate to cover the full range of expenses incurred as a result of an institutional delivery, including the cost of transportation and food. Related to this, another study undertaken in Nepal showed that women did not dare demand an institutional delivery because of worries about the associated financial burden [18]. Similarly, significant out-of-pocket expenses for transportation and food for women and their accompanying persons were of concern in Laos [53].

### Perceptions regarding the quality of health services

In our study, a woman’s decision to give birth in a health facility was positively influenced by the 24-h availability of a midwife and the friendly and caring behaviour of health workers. In contrast, women, their family members and FCHVs were all concerned about the perceived limited competence of the midwives. Consistently, a study in Nepal reported locally voiced doubt about the ability of the midwives in managing deliveries [54]. Lack of confidence in midwives working in rural health facilities has also been reported by studies in Ethiopia [41] and Indonesia [35], where village midwives were perceived to be young and inexperienced whereas TBAs were considered to be more mature and caring.

Moreover, some participants in the present study were concerned about the state of the infrastructure and available equipment at birthing centres; they also specifically worried about the lack of a heating system, suitable accommodation and appropriate foods. Lack of medication and inadequate equipment at the health facility along with the absence of an operating theatre, X-ray machines and laboratories for blood testing were identified as reasons for bypassing nearer birthing centres in a quantitative study among mothers in the same area of Nepal [55]. Similarly, in another study in Nepal, the lack of competent health workers and equipment in rural birthing centres discouraged women from using these facilities [56].

### Strengths and limitations of the study

Unlike a facility-based study, this community-based study included mothers who had given birth at home, in local birthing centres, at more remote health facilities across the Chitwan district as well as at hospitals at the district headquarter. VDCs and participants in FGDs and IDIs were selected purposively to capture views across the full caste and socio-economic spectrum. The insights gained therefore reflect a broad range of experiences of women during pregnancy, birth and the post-partum period. Importantly, the study’s findings apply to Chitwan district, in particular more remote plain and hill areas; they cannot be taken to apply to other districts of Nepal that may be characterized by a different geographical terrain as well as different ethnic, socio-cultural and socio-economic conditions.

This study has attempted to capture the views and experiences of women, their family members and other community members including FCHVs, as well as the views and experiences of health professionals at different levels of the health system. The triangulation of these multiple perspectives helped to tease out enablers and barriers to institutional delivery, which may be used as a starting point for strengthening enablers and overcoming barriers. As has become apparent during the analysis, even within a defined geographical area, there is much heterogeneity in the experiences and views between locations and in relation to personal and family backgrounds.

All data collection and transcription was undertaken in the local language by RS, who is a native Nepali speaker from Chitwan district, with the help of Nepali students who were trained in taking notes. Translations from Nepali into English were undertaken by RS with the help of competent local colleagues. The coding frame was developed by two independent coders, and all stages of the analysis involved several researchers to limit subjective interpretation. Since identification of codes was done on few FDGs and IDIs, it is possible that some information has been missed. Data analysis was undertaken in English, which may have distorted some of the more nuanced statements; where necessary, RS did go back to the original recordings to ensure that participants’ views were adequately captured.

## Conclusion

In the recent past, Nepal has paid increasing attention to the need to raise institutional delivery rates. Notably, in Chitwan district, the health system has made much progress in terms of providing physical and financial access to birthing centres and other health facilities offering relevant care during pregnancy, childbirth and the post-partum period. At community level, people increasingly recognize the importance of institutional delivery, especially in relation to complicated obstetrical situations. Yet, the findings of this study have provided some insights into the reasons why many women and their families in rural areas of Chitwan still reject giving birth at health facilities with some concrete implications for policy and practice. Importantly, they suggest that there is a need to strengthen the infrastructure of birthing centres in terms of heating equipment and accommodation, as well as to improve delivery of care through SBA training of midwives and sufficient availability of medical equipment and supplies. Similarly, at least in hill areas, access to suitable transportation must be improved and may require additional financial incentives. Moreover, focusing only on the biomedical aspects of a safe delivery is insufficient in overcoming existing socio-cultural barriers. This study has provided deep insights into some of the values and traditions that shape expectations with respect to the time around birth. For example, ensuring a warm environment at the birthing centre is critical, and families should be able to obtain or prepare culturally appropriate “hot” foods for women who recently delivered a baby. Also, husbands and mothers-in-law should be involved in programmes to increase uptake of institutional delivery.

National incentive programmes should consider local facilitators for and barriers to institutional delivery and enhance demand as well as improve quality of services such as friendly and culturally sensitive care. The successes or failures of the attempts to overcome the barriers that currently discourage women from making use of health facilities for giving birth should be carefully researched to enable learning over time.

## Abbreviations

ANC: Antenatal care
ANM: Auxiliary nurse midwife
DPHO: District public health officer
FCHV: Female community health volunteer
FGD: Focus group discussion
IDI: In-depth interview
MMR: Maternal mortality ratio
NRs: Nepali rupees
TBA: Traditional birth attendant
VDC: Village development committee
